# Long-lasting solid lubrication by CNT-coated patterned surfaces

**DOI:** 10.1038/srep42873

**Published:** 2017-02-17

**Authors:** L. Reinert, F. Lasserre, C. Gachot, P. Grützmacher, T. MacLucas, N. Souza, F. Mücklich, S. Suarez

**Affiliations:** 1 Department of Material Science and Engineering, Chair of Functional Materials, Saarland University, 66123 Saarbrücken, Germany

## Abstract

The use of lubricants (solid or liquid) is a well-known and suitable approach to reduce friction and wear of moving machine components. Another possibility to influence the tribological behaviour is the formation of well-defined surface topographies such as dimples, bumps or lattice-like pattern geometries by laser surface texturing. However, both methods are limited in their effect: surface textures may be gradually destroyed by plastic deformation and lubricants may be removed from the contact area, therefore no longer properly protecting the contacting surfaces. The present study focuses on the combination of both methods as an integral solution, overcoming individual limitations of each method. Multiwall carbon nanotubes (MWCNT), a known solid lubricant, are deposited onto laser surface textured samples by electrophoretic deposition. The frictional behaviour is recorded by a tribometer and resulting wear tracks are analysed by scanning electron microscopy and Raman spectroscopy in order to reveal the acting tribological mechanisms. The combined approach shows an extended, minimum fivefold longevity of the lubrication and a significantly reduced degradation of the laser textures. Raman spectroscopy proves decelerated MWCNT degradation and oxide formation in the contact. Finally, a lubricant entrapping model based on surface texturing is proposed and demonstrated.

With the increasing demand for lower energy consumption and CO_2_ emissions, the urge to reduce friction and wear to a minimal level, for example in automotive parts such as cylinder liners, piston rings or bearings in passenger cars, becomes increasingly important^1^. Introducing surface textures in materials has proven to be effective at lowering the coefficient of friction (COF) by reducing the real area of contact and by trapping wear debris and thus reducing the ploughing component of the COF^2^,^3^.

Out of various techniques to create well-defined surface patterns such as honing, micro coining or lithography, laser surface texturing (LST) and in particular direct laser interference patterning (DLIP) are suitable approaches^4^,^5^,^6^. In case of DLIP, interference fields produced by several coherent high-power laser sub-beams melt and shift material according to temperature- and thus surface tension gradients. This produces periodic patterns composed of different interference geometries such as line-, dot- or even lattice-like textures with typical feature sizes in the order of microns- down to several sub-microns, depending on the material^4^.

Studies on the tribological behaviour of DLIP-processed metallic surfaces have shown a significant reduction in friction and wear^2^,^3^. In addition to the effects caused by a reduced contact area and trapping of wear debris, the depressions of a textured surface may act as reservoirs for liquid or even solid lubricants, thus supplying the loaded contact with lubricants if necessary^7^,^8^. This mainly enhances the longevity of the lubrication and therefore avoids premature failure of machine components.

As far as solid lubricants or protective coatings are concerned, graphite, MoS_2_, DLC or Carbon Nanotube (CNT) coatings, amongst others, are able to mitigate friction and wear in loaded contacts. CNTs have received special attention due to their shape, high aspect ratio, sp^2^-carbon hybridisation state and high flexibility. Several studies have proved the ability of CNTs to reduce friction and wear, for example when used as reinforcement phase in composites^9^,^10^,^11^,^12^,^13^,^14^, as protective film^15^,^16^,^17^,^18^, solid lubricant^15^,^17^,^19^,^20^ or lubricant additive^21^,^22^,^23^,^24^. Regarding their friction- and wear-reducing effects, CNTs are often linked to roller bearings^11^,^25^,^26^, effectively rolling and separating the rubbing surfaces. Dickrell *et al*.^26^ were able to prove differences in the frictional behaviour of CNTs as a function of their orientation on top of a surface. Often, the friction reduction of CNTs is also explained by their degradation, forming a lubricating carbonaceous tribolayer^11^,^16^. In this case, simulations estimate a contact pressure threshold between 1.5 to 2.5 GPa to deteriorate the CNTs^27^.

Despite the abovementioned advantages, laser surface texturing and solid lubricants/protective films also have some drawbacks. In particular, surface textures may be gradually destroyed by plastic deformation, especially at the asperities with high local contact pressures^2^,^3^. Furthermore, coatings may be removed from the contact area depending on the loading conditions, thereby diminishing surface protection^28^.

The present study attempts to overcome these weaknesses by combining both approaches, namely, lubrication and its storage in textured depressions thus enhancing the endurance and efficiency of both, as well as reducing the COF. In order to do so, it is decisive to be able to copy an underlying texture (or in general complex geometries) with a homogeneous coating thickness.

Regarding the fabrication of CNT coatings, several techniques have been applied so far, including spray^29^ and dip coating^30^, CVD^31^, drop casting^28^ etc. Among those, electrophoretic deposition (EPD) has been successfully identified as a suitable, straightforward and easily scalable technique to deposit CNT films on different materials like metals or ceramics^32^. EPD is based on the electrophoresis principle: the motion of charged particles in an electric field and the subsequent adhesion/physisorption of these particles onto an electrode substrate in a fluid medium. It allows for short processing times, no shaping constraints, and homogeneous as well as controlled depositions, compared to drop casting or spin coating for example^32^,^33^. Decisively, EPD allows for copying complex geometries and textured surfaces resulting in an efficient deposition of coatings with homogeneous thicknesses^32^. For the given reasons, this study focuses on the combination of DLIP and EPD-deposited, multiwall CNT (MWCNT) coatings in order to study the tribological performance.

A commercially available steel (AISI 316) will be used as substrate material because of its technological relevance and due to the experience of our research group with this material in terms of laser patterning and the related effects on microstructural and chemical changes in the material^34^. The tribological results will be carefully analysed in terms of the coefficient of friction and the corresponding wear tracks. The wear tracks are analysed in light of microstructural and chemical findings by SEM/FIB and Raman spectroscopy, which also allows correlating the structural state of the MWCNTs with the tribological results. The tribological behaviour of the MWCNT-coated structures will be contrasted with that of bare (unlubricated) structures to isolate corresponding effects. Finally, a model will be presented which aims at explaining the mechanisms of entrapping the solid lubricant in the texture depressions.

## Experimental Section

### Materials

Commercially available MWCNTs (purchased at graphene supermarket, diameter 50–85 nm, length 10–15 μm) and steel (austenitic stainless steel AISI 316L) with a mirror-polished surface finish and a size of 20 × 20 × 1 mm^3^ were used as starting materials.

### Laser texturing

A pulsed Nd: YAG laser (Spectra Physics, Quanta Ray PRO 290) with a pulse duration of 10 ns and a repetition rate of 10 Hz was used for the laser structuring of the polished steel substrates. The primary laser beam was split into two sub-beams with equal intensity using suitable beam splitters. An overlap of these beams on the sample surface results in a line-like interference pattern. Due to the absorption characteristics of steel, the third harmonic of the laser system at 355 nm and a laser fluence of 400 mJ cm^−2^ were selected. Both, the laser fluence and a structural periodicity of the interference pattern (line spacing) of 9 μm were chosen based on previous studies that show the highest homogeneity of the structures using these parameters^2^,^3^.

### Roughness measurement

The roughness of the polished steel samples and the laser textured samples was measured using a white light interferometer (Zygo New View 7300) equipped with a 3-D imaging surface structure analyser. The root mean square roughness of the polished steel reference and the laser textured sample was 30 nm and 300 nm, respectively.

### Fabrication of CNT films

#### CNT functionalisation

The main drawback in CNT processing is the strong van der Waals interactions that generate agglomerates. During EPD, for instance, larger (and heavier) particles need larger voltages to be deposited, due to an enhanced viscous drag. However, the use of larger voltages tends to further aggregate separated CNTs^35^. Deposition thus loses homogeneity and density and incorporates pores^35^. Furthermore, larger particles tend to flocculate and settle leading to gradient film thicknesses in vertical electrode configurations^33^. In the case of non-flat surfaces the deposited material cannot replicate the original surface, which may translate to an easier detachment of the coating.

For the given reasons and the fact that CNTs are quasi inert and therefore unreactive during EPD, a covalent functionalisation step has been performed as suggested elsewhere^36^. 3 g of CNTs were refluxed in 225 ml of mixed concentrated nitric (68%, 168.75 ml) and sulfuric (97%, 56.25 ml) acid at 130 °C for 30 minutes. This step leads to the creation of acidic sites on the surface of the nanotubes, improving CNT dispersion and stabilisation by conferring a negative charge to the surface of the nanotubes^36^ and thus aiding the EPD process by reducing the presence of agglomerates. Afterwards, rinsing steps with deionised water were carried out with a 2 μm pore size filter. Finally, the CNTs were dried in a ventilated oven at 100 °C for 3 h to allow for the evaporation of water.

#### EPD process

For the deposition process, the mirror-polished reference and the laser-textured steel plates were used as electrodes. The plates were cleaned to remove possible contaminants, organic or other, that could interfere with the correct deposition of the CNTs. The cleaning consisted in a sequence of 10 min ultrasound steps, each in cyclohexane, acetone and isopropanol alternated with deionised water rinsing, except for the last step, immediately followed by drying in pressurised air.

A 0.25 mg ml^−1^ CNT solution was prepared by mixing the functionalised CNTs and acetone in a homogeniser (WiseTis HG-15, Witeg–Germany) for 5 min and ultrasonicating the solution for another 10 min in a bath (Bandelin sonorex super RK 514 BH, 35 kHz–860 W)^37^. Afterwards, 3 ml of triethylamine (TEA) were added in order to assist in the deprotonation of the CNT acidic groups^38^. Finally, the negatively charged CNTs could be deposited onto the anode (anodic deposition).

The electrophoretic depositions were performed in a deposition cell at a constant voltage of 20 V for 10 min, maintaining an electrode separation of 1.3 cm. After the deposition, the electrodes were taken out of the solution, keeping the voltage for 5 min. This assures that the film does not crack during the extraction of the electrodes from the liquid and also helps in the drying process^39^.

#### Chemical characterisation and wear track analysis

After the deposition of the CNT layer on the steel substrate, tribological experiments were performed. Prior to and after the tribological experiments, Raman spectra were acquired using an inVia Raman microscope (Renishaw) with an excitation wavelength of 532 nm in order to study the quality of the CNTs and to investigate tribo-oxidational effects. The data was recorded using a grating of 2400 lines mm^−1^, a 50x objective (numeric aperture: 0.9) and a laser power of 2 mW. The optical elements of the Raman microscope used in this analysis produced a laser spot size of roughly 5 μm (full width at half maximum) and a spectral resolution of about 1.2 cm^−1^. Every area is measured at least three times at different spots for statistical back-up and the mean value including the standard deviation is plotted.

After the tribological measurements, the samples were imaged using scanning electron microscopy (Helios Nanolab 600, FEI) in order to investigate the wear tracks, so as to analyse the potential underlying friction and wear mechanisms.

#### Tribological Experiments

Tribological experiments under dry sliding conditions (no additional oil or grease) were conducted using a ball-on-disk tribometer (CSM Instruments) in linear reciprocating sliding mode with a stroke length of 0.6 mm, a maximum sliding speed of 1 mm/s and a normal load of 100 mN. The number of cycles was set to 500 and 10.000 cycles in order to study the run-in behaviour and the long-term stability of the fabricated samples. The counter body was an Al_2_O_3_ ball with a diameter of 6 mm. The stroke length was chosen short enough to allow for several measurements at different spots of one sample, but also long enough to cover 67 maximum positions of the laser texture per stroke, therefore providing sufficient statistics. The normal load produces a contact radius of about 12 μm and a contact pressure of 346 MPa for the given material pairing (alumina against steel), calculated with the Hertzian contact model^40^,^41^ (used mechanical properties of the materials can be found in the supplementary information). This means, that at least two maximum positions of the laser texture are in direct contact with the ball at any time of the measurement. The choice of the ball material was made to avoid any plastic deformation of the counter body. The ball is mounted on a cantilever with a stiffness of 0.7624 μN μm^−1^ and 1.1447 μN μm^−1^ in normal and tangential directions, respectively. During the experiment, the deflections of the cantilever in the horizontal and vertical direction are measured using optical fibre displacement sensors. Consequently, the normal and friction forces can be calculated thus resulting in the coefficient of friction. Temperature and relative humidity were kept constant at 20 ± 2 °C and 4% ± 1%, respectively. All tribological experiments were conducted in perpendicular sliding direction in relation to the produced line-like laser-texture. This is because of previous studies showing the most pronounced friction and wear reducing effects when using this configuration^2^,^3^. For every sample, three measurements were conducted and the mean value including standard deviation was built and plotted against the number of sliding cycles.

## Results and Discussion

### EPD coating

**Figure 1:**
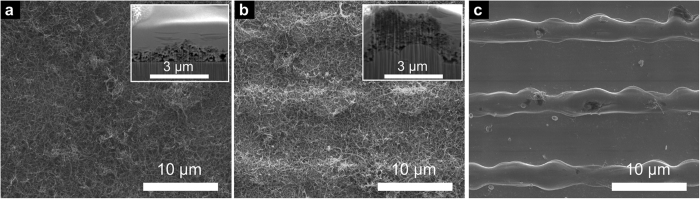
SEM micrographs of coated (**a**) untextured and (**b**) textured samples and (**c**) an as-textured sample. The structural periodicity of both laser textures is 9 μm. The insets in (**a**,**b**) show FIB-cross sections of the corresponding sample. There, the CNT-film can be seen between the steel substrate and the Pt protective film used for the cross section.

After laser-texturing (Fig. 1c), the samples were coated with CNTs by EPD. As expected from the negative surface charge of the functionalised CNTs, the deposition occurs at the anode and without any detection of hydrogen evolution during the deposition. Both untextured and textured samples could be homogenously coated (Fig. 1a,b). The deposited thickness is 1–2 μm, measured at different spots of either sample type by FIB-cross sections. Furthermore, the cross-sections (insets of Fig. 1a,b) show that the CNT film follows the surface’s profile. Finally, small CNT agglomerates are found within the coatings, possibly a consequence of the stated van der Waals interactions. Nonetheless, the entire surface is covered with disaggregated CNTs, providing a consistent surface coating.

### Frictional behaviour

**Figure 2:**
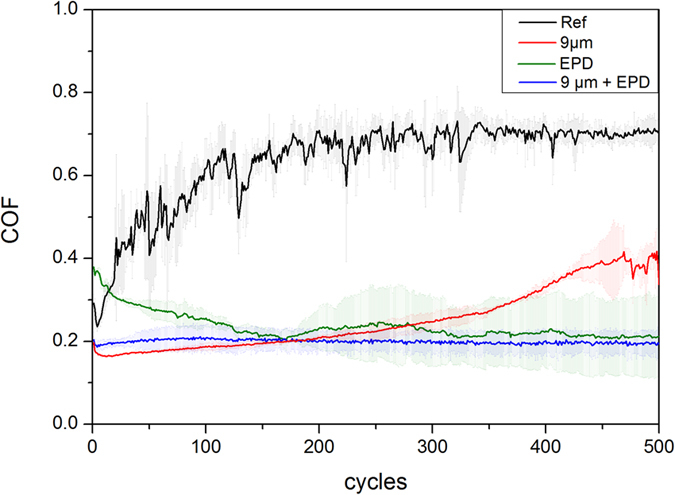
Temporal evolution of the dynamic COF (of one forward and backward sliding motion) plotted as a function of the number of sliding cycles (500 cycles) for the polished steel reference (Ref), textured, coated reference (Ref + coated) and textured + coated sample.

The different sample sets were compared in terms of the temporal evolution of the coefficient of friction (COF), which can be seen in Fig. 2 for a maximum of 500 sliding cycles.

The COF of the reference increases during the first 200 sliding cycles from 0.25 to roughly 0.7. After 200 sliding cycles, the COF remains stable at 0.7, thus reaching steady state conditions. The described behaviour is typical for pure metals and has already been extensively investigated elsewhere^42^. In the beginning of the experiment, only few single asperities of the ball and the substrate are in contact with each other thus generating a small contact area and consequently, a small COF. The increasing COF can be explained by the increasing real contact area, which is generated by wearing off said asperities and also by increasing the indentation depth of the ball into the substrate. However, the development of the COF appears to be very unstable for the reference compared to the other samples. The formation and disintegration of wear particle agglomerates might be responsible for this. The nature of those wear particles and the possible formation of an oxidic layer will be examined later in this work by SEM and Raman spectroscopy. In general, the steady state COF of the used material pairing for the reference correlates well with the literature^43^.

Regarding the textured sample, a continuous increase in the COF from 0.2 to 0.4 during the entire experiment can be noticed. It has already been shown, that the observed friction reduction is mainly attributed to a reduced real contact area^2^,^3^. This is reasonable, as the ball only gets in contact with the maximum position of the laser texture. The continuous increase of the COF during the experiment could be related to the steady increase in the real contact area due to a gradual removal of the surface texture. After 450 cycles, the COF fluctuates similarly to the reference, which might also be indicative of the destruction of the surface texture, therefore allowing the direct contact of the ball with wear particle agglomerates that are formed and broken up in a statistical manner. When regarding the COF of the ref + coated sample, a gradual decrease during 500 cycles from almost 0.4 to 0.2 is noted. Additionally, it is worth mentioning that the COF of the ref + coated sample is higher than the reference for the first 10 cycles. This may be explained by large amounts of entangled CNTs being shifted to the sides and ends of the wear track. The shifting and stacking of entangled CNTs requires a higher transversal force thus resulting in an even higher COF than the reference. Subsequently, a continuous supply of small amounts of CNTs seems to set in, acting as solid lubricant and reducing the COF^11^,^15^,^17^,^19^,^20^. Also, CNTs might be transformed to a lubricating carbon film^11^,^16^. Finally, the COF of the textured + coated sample appears constant and stable throughout the experiment at 0.2. This means that it shows influences of both friction reducing methods: laser-texturing at the beginning of the measurement and lubricant coating at the end of the measurement. Thus, this indicates a composite type behaviour of texture and solid lubrication. As for the other samples, a detailed wear track investigation and an investigation of the CNT lubrication activity might give a hint on the underlying friction mechanism, which will be discussed in detail later in the present study.

As the COF of the ref + coated- and the textured + coated samples is nearly identical after the first 150 cycles, it is reasonable to assume that this behaviour is mainly induced by CNTs present in the contact zones. This being stated, it is of interest to examine the long-term behaviour of these samples, as the textured + coated samples might provide CNTs to the contact area for a longer period of time.

**Figure 3:**
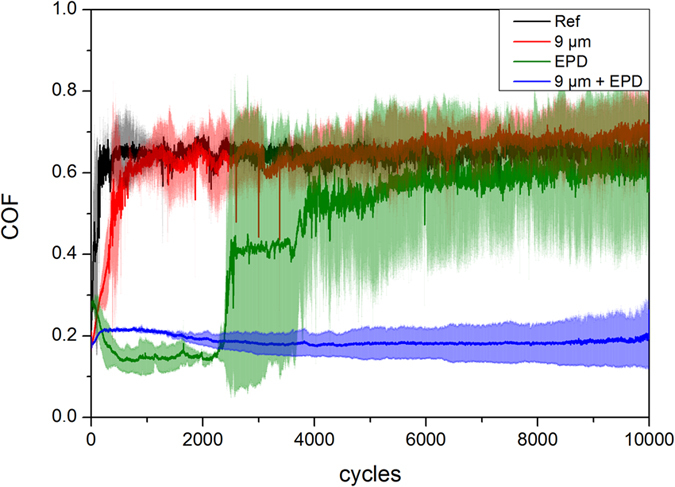
Temporal evolution of the COF plotted as a function of the number of sliding cycles (10000 cycles) for the polished steel reference (Ref), the textured, coated reference (Ref + coated) and textured + coated sample.

The temporal evolution of the COF at 10000 cycles provides us with information about the lifespan behaviour (Fig. 3). As described for the 500 cycles measurement, the reference clearly shows steady state behaviour after the first 200 sliding cycles for the already discussed reasons, approaching a COF of roughly 0.65.

The textured sample reveals an evolution of the COF very similar to that of the reference, yet only reaching steady state conditions after 1000 cycles. As already mentioned for the 500 cycles measurement, this might be a consequence of the ongoing destruction of the laser-texture, finally leading to the same contact conditions as the reference.

Looking at the ref + coated sample, the COF drops within the first 750 cycles from 0.35 to 0.15, remaining at this value for the next 1500 cycles. Subsequently, the COF sharply increases to 0.4 within 100 cycles, followed by a gradual approach to the reference value during the next 3500 cycles. This sharp increase might be a consequence of the degradation or disappearance of CNTs in the contact area, as described in the wear and Raman sections (3.3 and 3.4, respectively). It is noteworthy that the COF of the ref + coated sample shows a very high standard deviation after the first 2200 sliding cycles. This is due to individual measurements that have shown the observed sharp increase of the COF up to the reference value after different amounts of sliding cycles. All individual measurements have shown this sharp increase between 2200 and 4000 sliding cycles.

Finally, it should be pointed out that the textured + coated sample features the same low COF (0.2) for the entire 10000 sliding cycles. However, between 200 and 2200 cycles, the COF is higher than that of the ref + coated sample, which could be explained by the ability of CNTs to roll only on top of a flat, polished surface as already reported by Dickrell *et al*.^26^. This ability might be reduced in the case of the textured + coated sample, as the textured surface shows a much higher roughness of 300 nm compared to 30 nm of the reference (which is within the same order of magnitude as the CNT mean diameter), therefore hindering a rolling movement of the CNTs to a certain degree. However, the ongoing lubrication effect of the textured + coated sample for 10000 cycles is significant. A possible explanation for this would be the entrapping of solid lubricant (CNTs) by the laser-texture.

### Wear track

**Figure 4:**
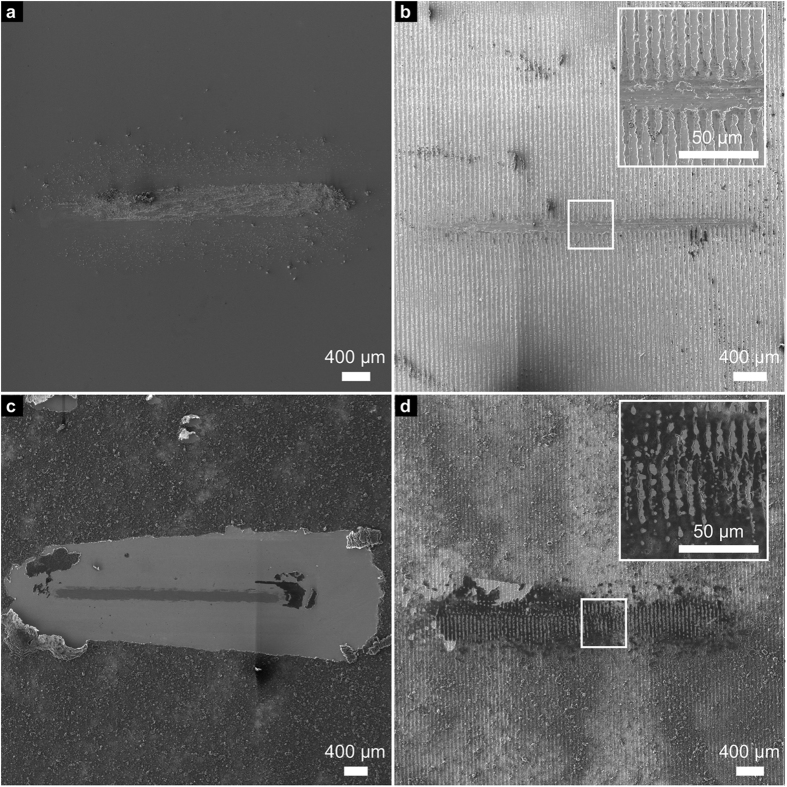
SEM micrographs of the wear tracks after 500 sliding cycles on (**a**) the reference, (**b**) the textured sample, (**c**) the ref + coated sample and (**d**) the textured + coated sample.

In general, a better understanding of the frictional behaviour of a tribological contact pair can be achieved by analysing the dominating wear mechanisms. In this context, it should be noted that different wear mechanisms usually act simultaneously during a tribological experiment. Therefore, only the dominating mechanisms are named and discussed in the following section. The wear tracks of all samples after 500 sliding cycles are depicted in Fig. 4 to allow for further discussion.

For the reference wear track, the dominating wear mechanism is ploughing, as can be seen in Fig. 4a. This is evident as the hardness of the counter body (Al_2_O_3_ ball) greatly exceeds that of the steel substrate. Wear particles are observed within and around the wear track, which might add an abrasive component in this respect. Also, the formation of a steady-state oxide layer might occur and contribute to the stabilisation of the COF^44^.

Regarding the textured sample after 500 cycles in Fig. 4b, the continuous increase of the COF during the experiment can be related to the steady increase in the real contact area due to a gradual removal of the surface texture. As in the reference wear track, the main acting wear mechanism is ploughing. However, it can be seen that the surface texture is not yet completely removed after 500 cycles and less severe wear is observed, compared to the reference. In general, less wear particles are found that are spread over the wear track. This might be a consequence of the reduced direct contact of the ball with wear particles as the minimum positions can trap wear debris, thus reducing the abrasive component^2^,^3^. As the laser-texture is still partially intact, the real contact area is still reduced compared to the reference and the COF is lower. The friction reducing effect within the first 500 cycles, induced by laser-textured steel surfaces has been published in previous studies^2^,^3^.

Regarding the corresponding wear track of the ref + coated sample (Fig. 4c), a clear shift of the CNT coating towards the end of the wear track can be observed. This supports the assumption that the high initial COF could be explained by shifting large amounts of entangled CNTs to the sides and ends of the wear track within the first sliding cycles. Subsequently, the wear track can be continuously supplied with small amounts of CNTs that are transferred from the ends of the wear track back to the contact area (grey area in the middle of the wear track), acting as solid lubricant and therefore reducing the COF again^11^,^15^,^17^,^19^,^20^. The CNTs, responsible for this lubrication effect, can be observed at both ends of the grey area and will be analysed in more detail within the chemical analysis. Despite that, no severe wear or wear particles can be noticed within the wear track compared to those of the reference and the textured sample.

Finally, the wear track of the textured + coated sample shows a very different appearance. After 500 cycles, the maximum positions are still present, even though they are a flattened, and the minimum positions are filled with CNTs as can be seen in Fig. 4d. This observation strongly supports the hypothesis of a solid lubricant entrapping caused by the laser-texture.

**Figure 5:**
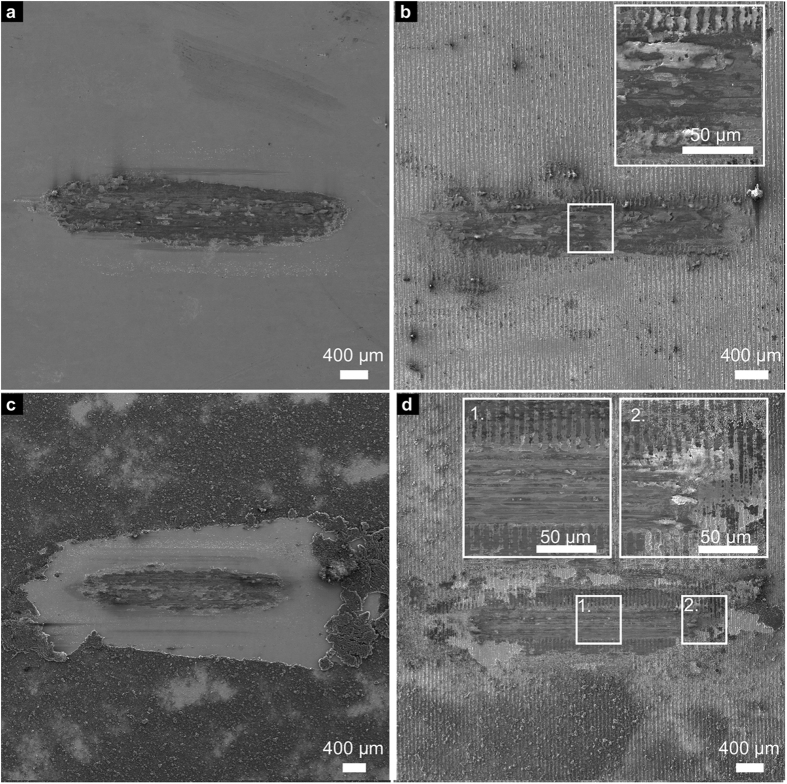
SEM micrographs of the wear tracks after 10000 sliding cycles on (**a**) the reference, (**b**) the laser textured sample, (**c**) the EPD coated sample and (**d**) the laser textured/EPD coated sample.

The wear tracks of the long-term measurements (10000 cycles) must be considered to understand the observed sharp increase of the COF after 2200 cycles in the case of the ref + coated sample, as opposed to the stability observed in the textured + coated sample. Therefore, SEM micrographs of the wear tracks after 10000 sliding cycles are depicted in Fig. 5.

For the reference, the behaviour of the COF as well as the dominating wear mechanism are already well explained by the previously described 500 cycle measurements. The only difference is the occurrence of much more severe wear as can be seen in Fig. 5a.

Regarding the wear track of the textured sample (Fig. 5b), it can be stated that the laser-texture has been completely removed within the contact area by ploughing, throughout exhibiting severe wear. Hence, a reduction of the real contact area can no longer be achieved and the COF behaves very similarly to the reference after the first 1000 cycles.

Comparing the wear track of the ref + coated sample after 10000 cycles in Fig. 5c, to that after 500 cycles in Fig. 4c, the additional appearance of severe wear is clearly noticeable. A reasonable explanation for this observation is the disappearance of the lubricating CNTs within the contact zone. The continuous removal of CNTs by the ball out of the contact zone as well as their absence at the ends of the severe wear area might be the reason for that. Hence, a direct contact of ball and substrate material is very likely to happen after the first 2200 sliding cycles, when a sharp increase of the COF can be observed. The slow increase of the COF for the next 3500 cycles (until it reaches the reference value) can be explained with an increasing real contact area between ball and steel substrate as well as individual measurements that show the described sharp increase of the COF at a later or earlier stage of the measurement.

When analysing the wear track of the textured + coated sample (Fig. 5d), different regions can be distinguished. In the middle of the wear track width, severe wear is observed with ploughing being the dominant wear mechanism. This region is very similar to the reference wear track after 10000 cycles. However, regarding the flanks of the wear track, an almost unworn laser-texture can still be noticed, storing CNTs in the minimum positions. It is therefore reasonable, that even after 10000 sliding cycles, the wear track is still supplied with CNTs that lubricate the system and keep the COF at a low value of 0.2. In comparison to the ref + coated sample, the CNTs are stored within the contact zone and don’t have to be applied from the surrounding area. Thus, the contacting surfaces are provided with CNTs for a considerably prolonged period of time. A differentiated discussion about oxide formation and structural state of the CNTs within the wear tracks is done in the following part of this study.

### Structural and chemical analysis

A deeper discussion of the COF evolution and the solid lubrication activity of the CNTs requires a detailed analysis of the tribochemistry as well as the structural integrity of the CNTs. In this study, the analysis is focused on the interpretation of the most relevant structural indicators obtained by Raman spectroscopy, namely: defect index (I_D_/I_G_), purity index (I_G′_/I_D_), and the G-band centre position (X_CG_).

**Figure 6:**
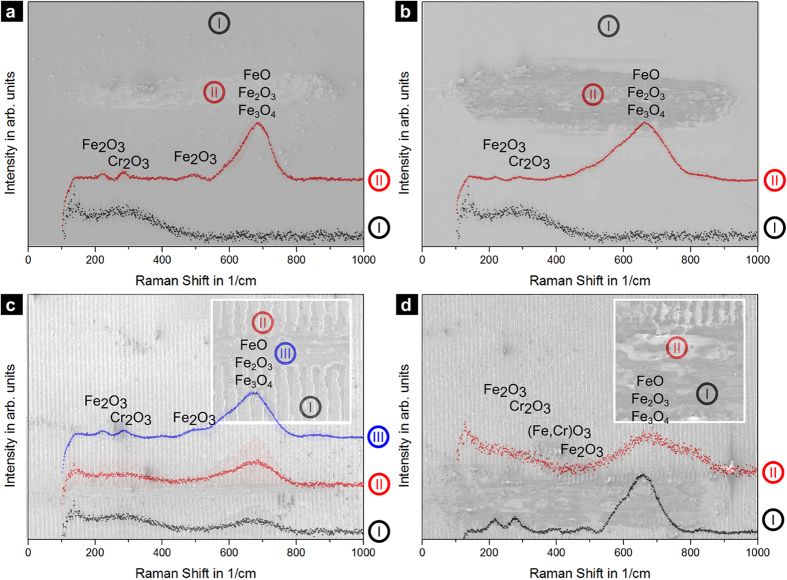
Raman spectra of the wear tracks at different spots of (**a**) the reference after 500 cycles and (**b**) 10000 cycles, and (**c**) the textured sample after 500 cycles and (**d**) 10000 cycles.

Figure 6 shows the electron micrographs and respective Raman spectra of the wear tracks after 500 and 10000 cycles for the reference and the laser-textured samples. In all the cases, regions of interest were defined based on their relevance to the study.

For the reference wear track after 500 cycles (Fig. 6a), the appearance of iron and chromium oxides (II) indicates that a tribologically-induced oxidation took place, due to the direct contact of the ball with the steel substrate under high local pressure^2^,^45^. The peak at 225 cm^−1^ corresponds to the α-Fe_2_O_3_ band, whereas the peak at 270 cm^−1^ is composed by a convolution of α-Fe_2_O_3_ and Cr_2_O_3_ peaks. The peaks at 380 cm^−1^ and 480 cm^−1^ are related to the occurrence of (Fe, Cr)O_3_ and γ- Fe_2_O_3_, respectively^45^,^46^,^47^. Finally, the peak with its maximum intensity at 660 cm^−1^ and a large full width at half maximum (Γ) is a convolution of FeO, Fe_3_O_4_ and Fe_2_O_3_ vibrational modes^45^. This observation consists of two different phenomena, the formation of a layer of oxidic nature and the development of oxidic wear particles during the experiment, which add an abrasive component. The latter is strongly related to the former, since the high local contact pressure applied breaks the oxide layer, generating wear particles that are included in the contact. These particles are subsequently embedded within the wear track (composed of the softer base metal) during the sliding motion, inducing a composite-type effect and contributing to the stabilisation of the COF.

In the case of the reference wear track after 10000 cycles (Fig. 6b), iron oxide is predominantly observed. This is reasonable as the COF of the reference is already in a steady-state condition after the first 200 cycles. Therefore, a change in the tribochemistry is rather unexpected. However, in comparison to the wear track after 500 cycles, the observed compositional changes in the oxide formation are probably due to a lack of chromium oxide detection based on its low volume fraction (compared to iron).

In the case of the textured sample after 500 sliding cycles (Fig. 6c), metal oxides are also found in the maximum (II) and minimum (I) positions outside of the severe wear track. This is explained by the thermally induced melting process during laser texturing and the consequent formation of an oxide layer^34^. Within the area of severe wear (III), the oxide peaks are considerably more pronounced, accordingly indicating a similar tribo-oxidation effect as observed on the reference material. Finally, for the wear track of the textured sample after 10000 cycles (Fig. 6d), no significant change in the oxidation behaviour can be observed compared to the short-term measurement. This leads to the conclusion, that the change in the COF (at almost 1000 cycles) in the specific case of the textured sample is exclusively generated by the increment in the real contact area due to wear occurring at the maximum positions. Interestingly, certain areas (I) can be found with pronounced oxide formation, whereas other regions (II) show less oxide formation. This might be a consequence of the on going ploughing mechanism which reorders oxides and bare metal in a stochastic manner.

**Figure 7:**
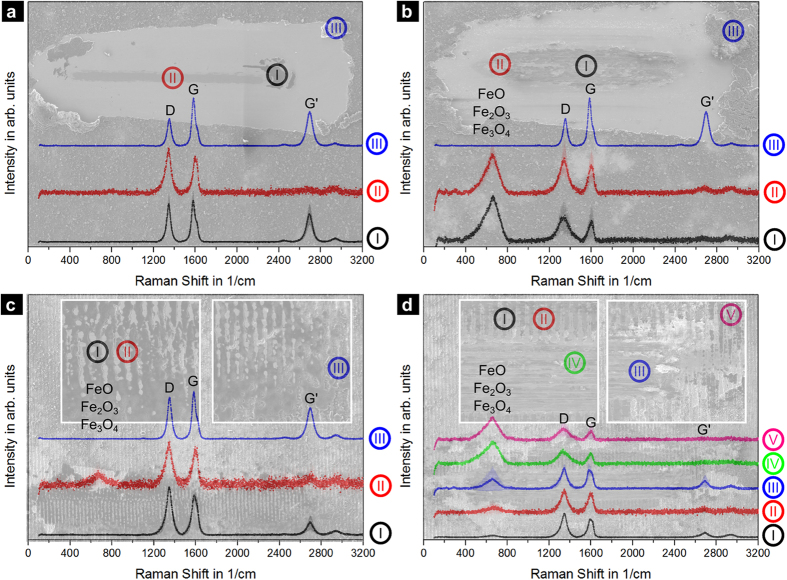
Raman spectra of the wear tracks at different spots of (**a**) the ref + coated sample after 500 cycles and (**b**) 10000 cycles and (**c**) the textured + coated sample after 500 cycles and (**d**) 10000 cycles.

For the analysis of the CNT lubrication activity, electron micrographs and respective Raman spectra of the coated sample wear tracks after 500 and 10000 cycles are depicted in Fig. 7. In addition to the Raman analysis of the centre and the outside of the wear track, it is also important to analyse the shifted CNTs that pile-up at the end of the wear track since it may also play a role during the tribological experiment.

The EPD coated sample shows no formation of oxides after 500 cycles (Fig. 7a). This supports prior observations on similar systems, where the CNT coatings have been proposed as oxidation protective coatings for low-cycle friction conditions^28^. In this context, CNTs are able to efficiently separate the steel surface from the ball, acting as a roller bearing and thus reducing friction and wear^11^,^48^. However, in Fig. 7b, intense oxide peaks can be found within the centre (I) and the end of the wear track (II) after 10000 cycles. Their appearance can be straightforwardly correlated to the coating failing after around 2200 sliding cycles, where a sharp increase in the COF is noticeable. Specifically, as no surface separation is possible anymore, the oxidation protection induced by the interstitial CNTs ceases. This leads to the formation of an oxide scale that, due to the large contact pressure develops into oxidic wear particles that act as an abrasive third body component participating in the transition from mild to severe wear.

Raman spectra of the wear track of the textured + coated sample (Fig. 7c) show a weak oxide peak on the texture maximum positions (II) after 500 cycles. As already mentioned, this is an unavoidable feature of the laser texturing. On the other hand, as observed in the ref + coated sample, the structure within the wear track (I and III) does not show remarkable oxide peaks, likely due to the oxidation damping provided by the CNTs. When analysing the wear track after 10000 cycles (Fig. 7d), it can be observed that, the intensity of the oxide peaks correlates to the extent of the effective contact area between counterpart and sample, being the minimum the position with the lowest amount (I), follow by the surface maximum (II), the end of the severe wear track (III) and finally within the severe wear track (IV); where the effective contact area between counterpart and sample is the highest. Interestingly, the strongest oxide peaks are detected at the very end of the wear track (V). This might be due to the fact that the generated oxidic wear particles of the severe wear track are shifted to the very end and there stored in the minima.

In the case of the ref + coated and the textured + coated samples, aside from the discussion of their oxidation behaviour, the focus of the analysis is also placed on the structural state of the CNTs. Table 1 shows the most relevant structural indicators to allow for a direct comparison.

The shifted CNTs (a III) have been transported out of the contact zone in the beginning of the measurement, showing almost identical defect and purity indexes as the reference state. Thus, they can be excluded from any tribological activity. Since these CNTs are out of the contact zone they do not exhibit any damage and retain their original structural integrity even after 10000 cycles (b III). CNTs found at the centre (a II) and end of the wear track (a I) show an increase in the I_D_/I_G_ ratio and a decrease in the purity index, indicative of CNT degradation. Comparing both regions, degradation seems to be significantly more pronounced in the centre (a I). This is due to the fact that this zone is situated where the maximum relative velocity is reached between the sample and the counterpart and the most severe mechanical and thermal stresses are expected. Specifically, the centre (a II) is located within the direct tribological contact zone, whereas the end (a I) would act as supplementary CNT storage. Although the shifted CNTs (a III) and the CNTs at the end of the wear track (a I) maintain the position of their G band, those present at the centre (a II) show an up-shifting to roughly 1600 1/cm.

This up-shifting has been thoroughly discussed in the literature by Ferrari and co-workers^49^ and corresponds to a transient state of the CNTs (clustering of the affected graphitic structure) towards nano-crystalline graphite. This is enclosed in a three-stage phenomenological model proposed by them, which analyses the transition from a graphitic-like to an amorphous-like structure (predominantly sp^3^-hybridisation) as function of the G-band centre position (X_CG_).

When observing the descriptive indexes for the ref + coated sample after 10000 cycles, an up-shift in the G band towards 1600 1 /cm is observed for CNTs within the centre (b I) and the end of the wear track (b II). The transient state is reached at the end of the wear track (b II) as well, inferring an accumulated CNT degradation towards a disordered state.

Regarding the textured + coated sample after 500 cycles, all measured regions show a degradation of the CNTs compared to the reference state CNTs. Particularly, those regions within the wear track (c I and II) have a defect index higher than at the end of the wear track (c III). However, the degradation in the interior regions is not as pronounced as in the centre (a II) of the ref + coated sample. This might be a consequence of the ongoing transfer of CNTs between maxima and minima during the experiment, thus exchanging the tribo-active CNTs more frequently. It should be kept in mind that the measured Raman spectra present a mean value of all the CNTs measured (for example degraded CNTs might be stacked upon intact CNTs in the minima). Interestingly, the G band position of the CNTs stored within the minima (c I) lies within the first stage of the amorphisation trajectory described in the Ferrari model. Then, it is reasonable to assume that those CNTs would sequentially be driven towards the contact zone as the experiment carries on. Thus, it becomes evident that the transition towards amorphous nanocarbons seems to be detrimental for the solid lubrication effect. However, the exchange between minima and maxima might lead to a general prolonged lifetime of the involved CNTs, as always just a small part of the involved CNTs is in a direct tribological contact. After the long term tests (d I–d V), all the analysed regions show similar Raman indices, each lying at the end of the first stage of amorphisation.

### Mechanism of entrapping solid lubricant

**Figure 8:**
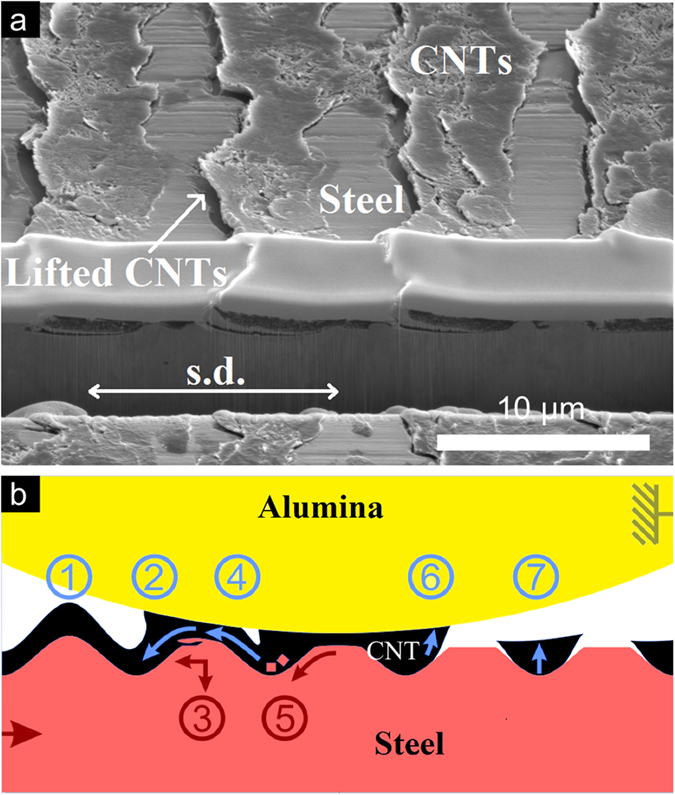
FIB cross-section of the wear track centre of (**a**) the textured + coated sample after 500 sliding cycles and (**b**) a schematic illustration, representing the acting mechanisms during the tribological experiment.

As both the wear track and Raman analyses correlate perfectly with the idea of a solid lubricant entrapping by the laser texture, the following part is focused mainly on this trapping mechanism and provides a schematic model to understand the frictional behaviour of the system. In Fig. 8, a high magnification SEM micrograph of a FIB cross-section within the wear track of the textured + coated sample after 500 sliding cycles (a) is depicted and correlated to a schematic draft of the mechanisms acting during the sliding motion (b).

In b1, the intact, coated laser texture is depicted. As soon as the ball gets into contact with the coating, CNTs are partially transferred to the alumina ball by adhesion (b2). This leads to the shifting of the CNTs towards the minima. At the same time, pressure is applied to the maxima of the laser-texture throughout the coating, resulting in a plastic deformation and a shearing-off of metallic material (b3). Due to adhesion effects, some CNTs are dragged towards the contact area (b4), preventing the direct contact of the alumina-ball with the steel substrate and therefore reducing the COF. Furthermore, the laser-texture prevents the full transfer of CNTs by storing most of the particles within the minima (b4-b5). In addition to the CNT storage capabilities of the minima, also metallic or oxidic wear particles could be partially stored (b5), therefore reducing abrasive wear. Finally, a slight lift of the stored CNTs mainly on one side of the maxima can be noticed (a). This is due to the mentioned adhesion between the alumina ball and the stored CNT particles during the sliding motion (b6), and elastic recovery of the compressed CNT volume after the stroke (b7). In the FIB cross-section (a), the last sliding cycle direction of the experiment was from left to right. Due to this observation and the ongoing lubrication, it can be expected that the protruding CNTs in the minima (b7) will be redragged into the direct contact zone of the maxima as soon as the sliding direction is inverted. These mechanisms can be well correlated to the lower COF compared to the ref + coated sample within the first 150 sliding cycles, as no additional transversal force for the CNT shifting is generated. The CNTs of the textured + coated sample (that were stacked and transferred out of the wear track in the ref + coated sample) are now stored in the minima, thus acting as a lubricant reservoir. In addition to a reduction of the real contact area by laser-texturing, the maxima are lubricated by the CNTs derived from the minima. Apart from milder wear than the textured sample, a reduced oxide formation was also noticed by Raman spectroscopy.

Summarizing, the combination of both methods, laser texturing and EPD coating, is found to overcome individual limitations by complementing one another. While the laser-texture provides the system with a certain load carrying capacity and lubrication storage, the CNT coating prevents its degradation, oxidation and acts as a long lasting solid lubricant.

## Conclusions

In the present work, CNT coating and laser texturing of steel surfaces are combined in order to act synergetically in terms of reducing friction and wear. The laser textures are perfectly copied by the CNT coating using EPD as coating technique. A tribological comparison to laser-textured/uncoated surfaces, untextured/CNT-coated surfaces and untextured/uncoated references is conducted. The following statements refer to a comparison with an untextured/uncoated reference:

1. **≤1000 sliding cycles:** Frictional reduction in the case of a **textured/uncoated sample**, which is accompanied by a gradual degradation of the surface texture.
2. **≤2200 sliding cycles:** Frictional reduction by a factor of four in the case of **untextured/coated sample** until the CNTs are dragged out of the contact zone and the COF increases.
3. **≥10000 sliding cycles:** Stable frictional reduction by a factor of three in case of **textured/coated sample**.

The slightly less pronounced frictional reduction of point 3 compared to point 2 can be explained with a higher roughness of the textured/coated sample (rms of 300 nm compared to 30 nm), thus hindering the CNT rolling movement and reducing the dimension of the lubricating effect. Furthermore, a direct correlation of the CNT degradation in a tribological contact with an already known three-stage phenomenological model (transition from graphitic-like to amorphous-like structures) is established using Raman spectroscopy. It is found, that the combination of laser texturing and CNT-coating slows down the degradation process of the CNTs within a tribological contact and also the formation of oxidic wear particles. Finally, the significant extension of the lubricant longevity in point 3 is related to a synergistic effect of CNTs and laser textures and can be exemplified by a lubricant entrapping model, which is proposed and demonstrated.

## Additional Information

**How to cite this article**: Reinert, L. *et al*. Long-lasting solid lubrication by CNT-coated patterned surfaces. *Sci. Rep.* **7**, 42873; doi: 10.1038/srep42873 (2017).

**Publisher's note:** Springer Nature remains neutral with regard to jurisdictional claims in published maps and institutional affiliations.

## Supplementary Material

Supplementary Information

## Figures and Tables

**Table 1:** Descriptive parameters obtained by Raman spectroscopy of the coated samples, measured at different positions in the wear track.

Sample	Cycles	Position	I_D_/I_G_	I_G′_/I_D_	X_CG_ (1/cm)	Description
Initial CNT			0.595	1.294	1583.2	Reference state
Ref + coated	500	a I	0.918	0.735	1585.8	End of wear track
		a II	1.278	0.156	1602.8	Centre of wear track
		a III	0.563	1.273	1585.4	Shifted CNTs
	10000	b I	1.167	0.226	1598.4	Centre of wear track
		b II	1.266	0.222	1599.0	End of wear track
		b III	0.564	1.272	1587.4	Shifted CNTs
Textured + coated	500	c I	1.195	0.274	1591.7	Min–inside
		c II	1.121	0.292	1597.8	Max–inside
		c III	0.876	0.751	1586.7	End of wear track
	10000	d I	1.227	0.203	1598.9	Min–inside
		d II	1.129	0.255	1598.3	Max–inside
		d III	1.175	0.402	1592.1	End of severe wear
		d IV	1.202	0.311	1597.0	Centre of wear track
		d V	1.235	0.210	1598.3	Min–End of wear track

(Min–inside) stands for minimum within wear track, (Max–inside) stands for maximum within wear track, and (Min–End of wear track) stands for minimum at the end of the wear track.
