# Supplementary material for: Long-lasting solid lubrication by CNT-coated patterned surfaces
Source: Sci Rep. 2017 Feb 17;7:42873. doi: 10.1038/srep42873 (PMC5314357; doi:10.1038/srep42873)
Supplement: Supplementary Information [file srep42873-s1.doc]

CNT coatings as the icing on laser textures - Entrapping solid lubricant to last longer (*supplementary info*)

L. Reinert^1^*, F. Lasserre^1^, C. Gachot^1^, P. Grützmacher^1^, T. Maclucas^1^, N. Souza^1^, F. Mücklich^1^, S. Suarez^1^

^1^ Department of Material Science and Engineering, Chair of Functional Materials, Saarland University, 66123 Saarbrücken, Germany

***** Correspondence: l.reinert@mx.uni-saarland.de; Tel.: +49-681-302-70544; Fax: +49-681-302-70502

Table 1: Mechanical properties of AISI 316-L stainless steel, Alumina and MWCNT ^1,2,3,4,5^

| Material | E-Modulus | Yield Strength (Compressive strength for Alumina) | Hardness |
| --- | --- | --- | --- |
| AISI 316-L | 200 GPa | 200 MPa | 2.4 GPa |
| Alumina | 380 GPa | 2.5 GPa | 16 GPa |
| MWCNT | 1 TPa | 30 GPa | - |

1. Rosenkranz, A. et al. Oxide Formation, Morphology, and Nanohardness of Laser-Patterned Steel Surfaces. Adv. Eng. Mater. 10.1002/adem.201400487 (2015).

2. Yu, M. Strength and Breaking Mechanism of Multiwalled Carbon Nanotubes Under Tensile Load. Science. 287, 637–640 (2000).

3. Salvetat, J.-P. et al. Mechanical properties of carbon nanotubes. Appl. Phys. A Mater. Sci. Process. 69, 255–260 (1999).

4. Datasheet Alumina from Friatec, retrieved on 17.08.2016 at http://www.friatec.de/content/friatec/de/Keramik/FRIALIT-DEGUSSIT-Oxidkeramik/Downloads/downloads/FA_Bauteile-aus-AL2O3-und-ihre-Nachbearbeitung.pdf

5. Datasheet AISI 316-L from Deutsche Edelstahlwerke, retrieved on 17.08.2016 at http://www.friatec.de/content/friatec/de/Keramik/FRIALIT-DEGUSSIT-Oxidkeramik/Downloads/downloads/FA_Bauteile-aus-AL2O3-und-ihre-Nachbearbeitung.pdf
